# Spatial metabolomics on liver cirrhosis to hepatocellular carcinoma progression

**DOI:** 10.1186/s12935-022-02775-9

**Published:** 2022-11-24

**Authors:** Michelle Junyi He, Wenjun Pu, Xi Wang, Xiaoni Zhong, Dong Zhao, Zhipeng Zeng, Wanxia Cai, Jiayi Liu, Jianrong Huang, Donge Tang, Yong Dai

**Affiliations:** 1 Clinical Medical Research Center, The Second Clinical Medical College of Jinan University, Shenzhen People’s Hospital, No. 1017 Dongmen North Road, Shenzhen, 518020 China; 2 Department of Biology, Department of Brain and Cognitive Sciences, Massachusetts Institute of Technology, Cambridge, MA 02139 USA; 3 The First Affiliated Hospital, Jinan University, Guangzhou, 510632 China; 4 The First Affiliated Hospital (Shenzhen People’s Hospital), Southern University of Science and Technology, Shenzhen, 518055 China; 5 Department of Nephrology Center, Department of Liver Transplant Center, The Third People’s Hospital of Shenzhen, The Second Affiliated Hospital of Southern University of Science and Technology, Shenzhen, 518100 Guangdong China; 6 Guangxi Key Laboratory of Metabolic Disease Research, Central Laboratory of Guilin, 924St Hospital, Guilin, 541002 China

**Keywords:** Spatial metabolomics, Liver cirrhosis, Hepatocellular carcinoma, Disease progression, Amino acid metabolism

## Abstract

**Background:**

Hepatocellular carcinoma (HCC) is one of the deadliest cancers and is mainly developed from chronic liver diseases such as hepatitis-B infection-associated liver cirrhosis (LC). The progression from LC to HCC makes the detection of diagnostic biomarkers to be challenging. Hence, there have been constant efforts to improve on identifying the critical and predictive changes accompanying the disease progression.

**Methods:**

In this study, we looked to using the mass spectrometry mediated spatial metabolomics technique to simultaneous examine hundreds of metabolites in an untargeted fashion. Additionally, metabolic profiles were compared between six subregions within the HCC tissue to collect spatial information.

**Results:**

Through those metabolites, altered metabolic pathways in LC and HCC were identified. Specifically, the amino acid metabolisms and the glycerophospholipid metabolisms experienced the most changes. Many of the altered metabolites and metabolic pathways were able to be connected through the urea cycle.

**Conclusions:**

The identification of the key metabolites and pathways can expand our knowledge on HCC metabolic reprogramming and help us exam potential biomarkers for earlier detection of the malignant disease progression.

**Supplementary Information:**

The online version contains supplementary material available at 10.1186/s12935-022-02775-9.

## Background

Liver cancer is currently one of the most fatal malignancies worldwide and poses great challenges to early diagnosis and prognosis due to its heterogeneity. The most common form of liver cancer is hepatocellular carcinoma (HCC), which accounts for greater than 75% of the cases [21]. HCC usually arises from chronic liver inflammation that can be caused by a variety of risk factors such as hepatitis B and/or C infections, alcohol abuse, obesity, diabetes mellitus, etc. [1, 15, 18, 29]. Particularly, the progression from chronic hepatitis B infection to liver cirrhosis then to HCC is one of the most common ways of disease occurrence.

Hepatitis B is a life-threatening liver infection caused by the hepatitis B virus (HBV) and is transmittable through body fluid. The infection causes alterations of many cellular processes and leads to scarring of the liver, both of which greatly increase the chance of liver cirrhosis and liver cancer. Such liver injury can lead to changes in cell signing, DNA repair, apoptosis, etc., and results in the accumulation of reactive oxygen species, and/or oncogene activation [29]. These cellular level changes often time worsen into loss of cell cycle and senescence control, dysregulations of apoptosis and NF-κB pathway, etc. that reflect HCC progression [11, 13, 16, 29, 37]. Thus, researchers have been trying to map out the cellular causes of the progression from hepatitis B infection to liver cancer. However, the progression of the disease usually takes various pathways and different paces in individual patients and can be affected by genetic susceptibilities. Therefore, the inter-and intra-personal heterogeneity of the disease has made the identification of the exact mechanisms very challenging.

Recently, with the advancement of new technologies, the identification of HCC-related biomarkers has become a promising way to decode the disease progression mechanisms. Specifically, there has been increasing interest in metabolomics, or the study of metabolites. Global metabolomics with liquid or gas chromatography has revealed alteration in various metabolic pathways such as the TCA cycle, glycolysis, lipid synthesis, etc. [9]. Noticeably, metabolites such as Glypican-3 [3], monounsaturated fatty acids, and the ratio between polyunsaturated fatty acids omega-3 and omega-6 [10] were found to be important biomarkers for the progression of liver cancer. However, global metabolomics only looks at the average of the tissues. Furthermore, most of the previous studies regarding liver cancer look at blood samples for finding key metabolites. These methods have limitations as they may omit important regional variations of the metabolites that mark liver disease progression or the boundaries of diseased tissues.

Now, with the development of the mass spectrometry imaging (MSI) technique, spatial metabolomics emerges as a promising direction of study. Such a study takes into account the spatial variation of metabolites in the tumor microenvironment and therefore can be useful in detecting the metabolic biomarkers of liver cancer. As spatial metabolomics is still a relatively new field of study, there has not been any systematic study that analyzes the alterations of metabolites distribution and abundance in liver diseases tissues. Hence, in this study, we used the air flow-assisted desorption electrospray ionization mass spectrometry imaging (AFADESI-MSI) technique to examine altered metabolites and metabolic pathways in liver cirrhosis and HCC tissues compared to the healthy control. AFADESI-MSI was able to simultaneously detect hundreds of metabolites in situ with high sensitivity. Therefore, through this experiment, we were able to identify key metabolites that change continuously as the disease progress.

## Methods

### Tissue collection and preparation

**Fig. 1:**
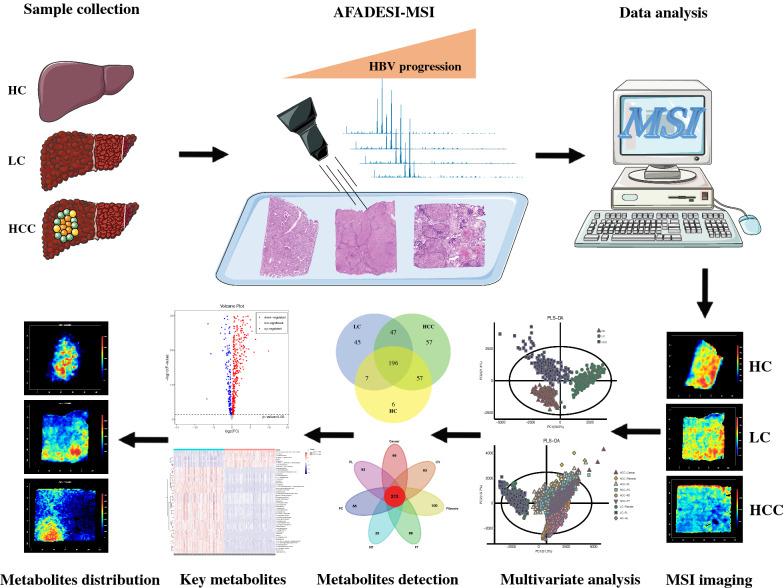
Schematics of using the AFADESI-MSI method to detect spatial distributions of metabolites in the healthy control (HC), HBV relative liver cirrhosis (LC) and liver cancer (HCC) samples

All the sample tissues were surgery remnants acquired at Shenzhen People’s Hospital in 2021. This study was approved by the ethics committees of the Shenzhen People’s Hospital (LL-KY-2021723). Informed consent about the study was collected from all participants. The flow chart of the experiment can be found in Fig. 1. Clinical data of the liver cirrhosis (LC) and HCC patients listed in Table 1 were collected one day before the operation. Sample tissues from the three different conditions of livers were collected, frozen, and sectioned to prepare for Mass Spectrometry Imaging (MSI). The three samples were cancerous tissue, liver cirrhosis, and healthy liver tissue. Following the acquisition of the samples, tissues were flash-frozen in liquid nitrogen and stored at − 80 °C until sectioning. The day prior to sectioning, samples were moved from − 80 to − 20 °C to de-freeze overnight. Then, sectioning was carried out using the Leica CM1950 cryostats. The sample slices were thaw-mounted to superfrost plus positively charged slides (Thermo Fisher) and stored at − 80 °C until the MSI experiment. One of the tissue sections from each sample was stained with hematoxylin and eosin (HE) staining solution for histological analysis.

**Table 1:** The clinical data for liver cirrhosis and HCC participates

Measurements	LC	HCC	Average	Reference^a^	Change
TP (g/L)	52.70	58.10	55.40 ± 2.70	63.00–79.00	↓
ALB (g/L)	34.80	39.80	37.30 ± 2.50	35.00–50.00	–
GLO (g/L)	17.90	18.30	18.10 ± 0.20	20.00–35.00	↓
A/G	1.94	2.17	2.06 ± 0.12	1.10–2.50	–
PA (mg/L)	83.00	152.00	117.50 ± 34.50	150.00–350.00	↓
TB (μmol/L)	41.30	194.80	118.10 ± 76.75	1.71–20.50	↑
GLD (U/L)	9.32	7.00	8.16 ± 1.16	< 7.00	↑
DB (μmol/L)	11.00	70.00	40.50 ± 29.50	< 5.10	↑
ID (μmol/L)	30.30	124.80	77.55 ± 47.25	3.40–12.00	↑
TBA (μmol/L)	140.00	136.60	138.30 ± 1.70	< 10.00	↑
ALT (U/L)	31.00	37.00	34.00 ± 3.00	7.00–55.00	–
AST (U/L)	36.00	52.00	44.00 ± 8.00	8.00–48.00	–
GGT (U/L)	20.00	33.00	26.50 ± 6.50	8.00–61.00	–
ALP (U/L)	71.00	52.00	61.50 ± 9.50	40.00–129.00	–
CHE (U/L)	3704.00	2987.00	3346.00 ± 358.50	8k–18k	↓
LDH (U/L)	216.00	933.00	574.50 ± 358.50	140.00–280.00	↑
PT (S)	18.70	23.60	21.15 ± 2.45	11.00–13.50	↑
PT% (%)	51.00	36.00	43.50 ± 7.50	100.00	↓
PT INR	1.58	2.15	1.87 ± 0.29	0.80–1.10	↑
APTT (S)	55.00	40.80	47.90 ± 7.10	21.00–35.00	↑
FIB (g/L)	1.53	1.54	1.54 ± 0.01	2.00–4.00	↓
TT (s)	18.10	21.70	19.90 ± 1.80	14.00–19.00	↑
AT III (%)	39.00	41.00	40.00 ± 1.00	80.00–130.00	↓
D-DIC (μg/mL)	0.22	> 20	10.11 ± 9.89	0.10–0.25	↑
AMON (μmol/L)	133.00	101.00	117.00 ± 16.00	11.00–32.00	↑
HBsAg	20.91 (+)	31.93 (+)	26.42 ± 5.51	N/A	N/A
HBsAb	0 (−)	0.99 (−)	0.50 ± 0.50	N/A	N/A
HBeAg	0.377 (+)	0.04 (−)	0.21 ± 0.17	N/A	N/A
HBeAb	1.54 (−)	0.01 (+)	0.78 ± 0.77	N/A	N/A
HBcAb	5.37 (+)	0.21 (−)	2.79 ± 2.58	N/A	N/A
Child–Pugh	9	13	11.00 ± 2.00	N/A	N/A
MELD	32	44	38.00 ± 6.00	N/A	N/A

Averages are expressed as mean ± SEM

TP, total protein; ALB, albumin; GLO, globulin; A/G, albumin/globulin ratio; PA, prealbumin; TB, total bilirubin; GLD, glutamate dehydrogenase; DB, direct bilirubin; ID, indirect bilirubin; TBA, total bile acid; ALT, alanine transaminase; AST, aspartate transaminase; GGT, gamma-glutamyl transferase; ALP, alkaline phosphatase; CHE, cholinesterase; LDH, lactate dehydrogenase; PT, prothrombin time; INR, international normalized ratio; APTT, activated partial thromboplastin time; FIB, fibrinogen; TT, thrombin time; AT III, antithrombin III; D-DIC, D-dimer; AMON, ammonia; HBsAg, hepatitis B virus surface antigen; HBsAb, hepatitis B virus surface antibody; HBeAg, hepatitis B virus e antigen; HBeAb, hepatitis B virus e antibody; HBcAb, hepatitis B virus core antibody; MELD, model of end stage liver disease

^a^Reference ranges may vary with patient’s sex, age, pregnancy, etc., and may be different depending on materials and methods used

### AFADESI-MSI

Both positive- and negative-ion mode Air Flow-Assisted Desorption Electrospray Ionization Mass Spectrometry Imaging (AFADESI-MSI) were performed using the AFADESI platform (Tscience, China) coupled with the Q-Orbitrap mass spectrometer (Thermo Fisher). The AFADESI platform replaces the original ion source and provides a high-rate airflow that assists the electrospray for improved ionization and ion collection. The propelled secondary ions were then collected and transferred to the Q-Orbitrap mass spectrometer. Based on the specific properties of the compounds, different metabolites may have higher chance of being detected in different ionization modes. In the case of most DESI-MSI, positive ionization mode is generally shown to have higher sensitivity and stability as the negative mode is prone to corona discharge [4, 26].

The spray solutions for both the positive- and the negative-ion mode were prepared by mixing acetonitrile and water (4:1, V/V). In addition, the solution for the positive-ion mode contained 0.1% formic acid. Prior to running through the mass spectrometer, the frozen samples were taken out of − 80 °C and were dried at room temperature inside the vacuum dryer for 30 min. Samples were then placed on an XY translation stage to enable continuously scanning of the sample line by line at a constant rate of Vx = 0.2 mm/s. The distance between each scanning line (Dy) was set to be 0.1 mm. The entire scanning area was 10 mm by 10 mm. The spray gas press was set at 0.6 MPa, the capillary temperature was at 350 °C, the spray gas flow rate was at 5 μL/min, and the extracting gas flow rate was at 45 L/min. Additional parameters of the MSI experiment can be found in Additional file 2: Table S1.

### Data analysis

Ion intensity across each position was outputted as raw data. All raw data files were converted into “.cdf” format, and data analysis was conducted using the custom-developed imaging software, MassImager [12], for image reconstruction. Spatial shrunken centroid clustering (based on K-Means clustering) was then performed to generate K-Means plot. These plots were compared to H&E staining images to extract the region of interest (ROI) and construct the MS profiles. Ions were compared to the online database HMDB (https://hmdb.ca/) and SMPD (https://www.smpdb.ca/) for metabolites identification (ppm < 5). Differential metabolites were identified based on Student’s t test and fold change analysis.

## Results

### Clinical characteristics of the participants

Clinical data of the LC and HCC patients were collected for comparison, and significant differences were shown between many of the patients’ measurements and the reference levels. Relevant clinical data for the patients are summarized in Table 1. Particularly, bilirubin and total bile acid levels were greatly increased, reflecting the patients’ compromised ability to break down bile. In addition, prothrombin time and INR also increased in both LC and HCC patients, suggesting a reduced production of blood-clotting proteins. Generally, there was a decrease in protein levels for both patients. Furthermore, almost all the observed changes were more severe in the HCC patient than the LC patient, showing a progression in liver disease severity. The Child-Pugh and MELD scores reflected such disease progression. Lastly, both the LC and HCC patients were tested for HBV infection, and both patients either had past or active infection shown by their positive antibody tests.

### Overall metabolic profiles of the tissue samples

**Fig. 2:**
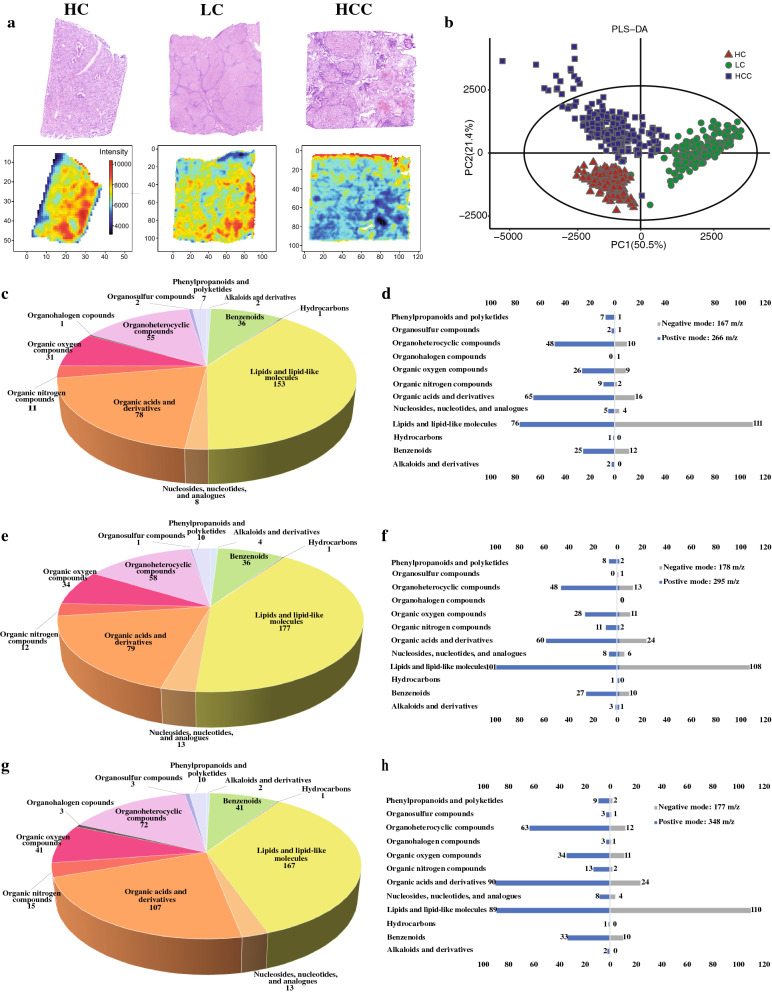
Total metabolites detected by AFADESI-MSI method in HC, LC and HCC tissue samples under positive ionization mode. **a** HE and MSI diagrams of HC, LC and HCC whole samples. **b** PLS-DA comparison of the AFADESI-MSI data. **c**, **e**, **g** Total metabolites detected by AFADESI-MSI method in HC, LC and HCC samples, respectively. **d**, **f**, **h** Differences between the detected metabolites under positive and negative ionization mode in HC, LC and HCC samples, respectively

AFADESI-MSI was performed on the sample tissues and the MSI profiles were constructed to analyze the global metabolic profiles. Under the positive ion mode, AFADESI-MSI was able to detect ions ranging from *m/*z 70–800. Both the H&E stain images and the example MSI diagrams revealed that all three liver tissues have considerable intra- and inter-sample heterogeneity. Figure 2a shows the overlay MS images of all the detected metabolites. Based on the differential regional intensities of the ions, all three samples have considerable intra-sample variations in terms of the spatial metabolic profiles. There appeared to be meaningful patterns in the overall distributions of metabolites that invited us to cluster the profiles. The k-mean diagrams in Additional file 1: Fig. S1e&f then display clustering results of the metabolites and reveal that the sample tissues can be separated into different regions, possibly representing different tissue types. From the MS images, it appeared that the three samples differed between each other, too. The partial least squares-determinant analysis (Fig. 2b) was able to separate the three tissue types (HC, LC, HCC) based on their differential metabolites. To make sense of the separation, the relative abundances of the different classes of metabolites were first examined. Overall, the compositions of the metabolites being detected in HC, LC, and HCC samples were similar, with lipids and lipid-like molecules, organic acids and derivatives, and organoheterocyclic compounds being the three most abundant types of metabolites (Fig. 2c, e, g). However, compared to the other two samples, lipid and lipid-like molecules had larger percentage among total metabolites in the LC sample (Fig. 2e) while organic acids and derivatives were found to have a larger abundance in the HCC sample (Fig. 2g). Therefore, the global metabolic profiles of the three samples suggest not only the regional diversity of liver tissues but also the metabolic differences between the three liver conditions.

To check the consistency of the results, negative ion mode data was compared. Generally, the relative abundance of each class of metabolite was consistent (Fig. 2d, f, h). Yet, it was also noticeable that the negative ion mode resulted in a lower sensitivity compared to the positive ion mode for all classes of molecules other than lipid and lipid-like molecule. Hence, positive ion mode data were used in later analyses.

### Identification of ROIs and differential metabolites within sub-regions

**Fig. 3:**
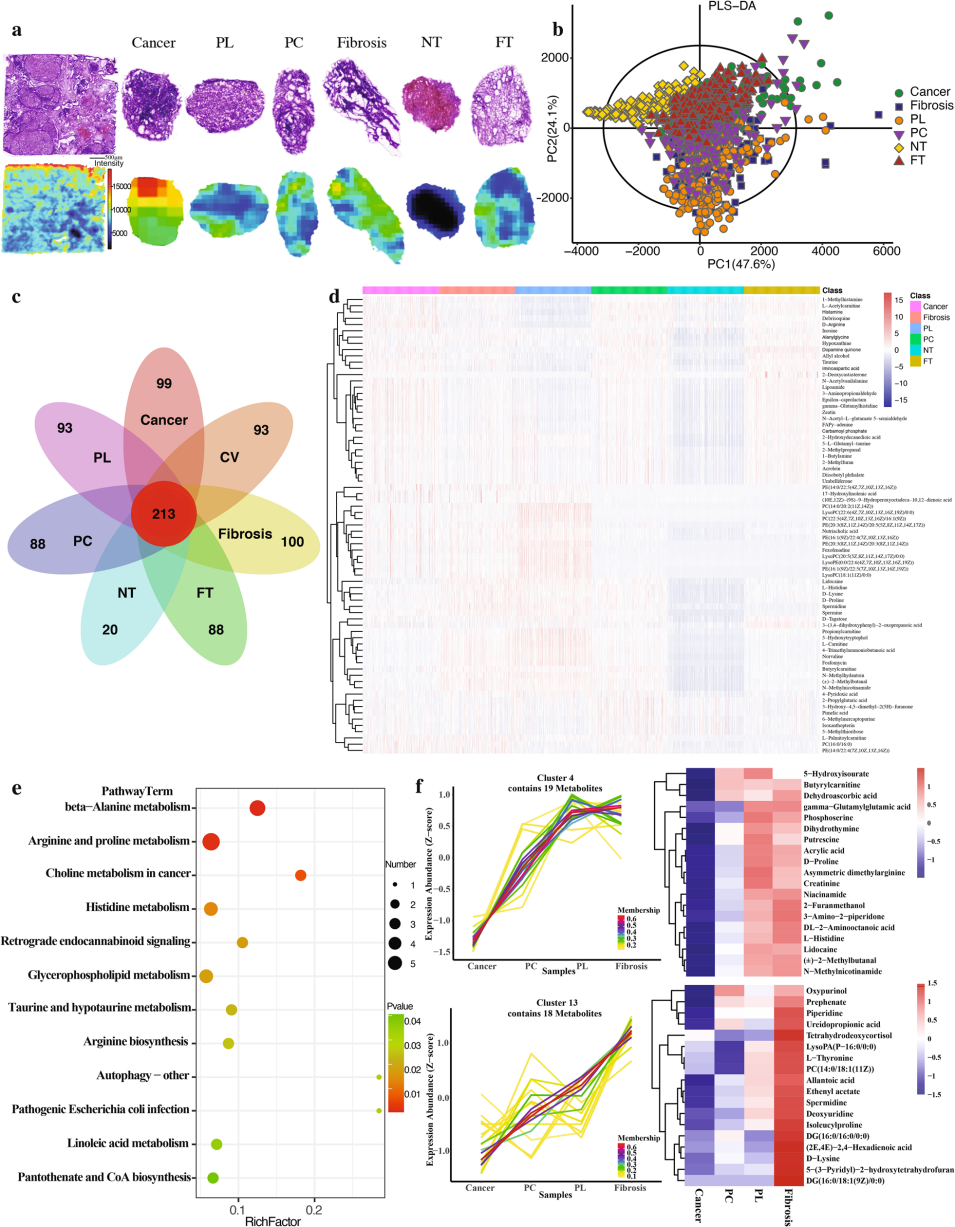
The alterations of metabolites’ spatial distributions in sub-regions of HCC sample under positive ionization mode. **a** HE and MSI images of different sub-regions in HCC including cancer, pseudo lobule (PL), pre-cancer (PC), fibrosis, necrotic tissue (NT), and fatty tissue (FT). **b** PLS-DA analysis for HCC sub-regions. **c** Number of metabolites detected from different HCC sub-regions. **d** Heatmap of significantly differentiated metabolites based on variable importance of projection > 1 (VIP > 1). **e** KEGG analysis of key altered metabolic pathways between the sub-regions of HCC. **f** Sample time series analyses of key metabolite expressions in all sub-regions of HCC

A total of six regions of interest (ROI) were identified by overlaying the H&E and the MSI images of the HCC sample. Their region-specific metabolic profiles were analyzed to reveal any key differential metabolites. Figure 3a displays the H&E stain and MSI images for the entire HCC sample and the zoomed-in images for the six ROIs: cancerous region, pseudo lobule (PL), necrotic tissue (NT), fibrosis, pre-cancer region (PC), and fatty tissue (FT). Particularly, the cancerous region and the necrotic tissue had the most distinct MSI profiles whereas the other regions had more comparable ion intensity patterns. The cancer region contained an overall abundance of metabolites while the necrotic region showed a lack of metabolites. The results for negative-ion mode MSI are shown in Additional file 1: Fig. S2. Under this imaging mode, the heterogeneity within the sub-regions is less visible, but there are still detectable variabilities of the metabolite distribution patterns. Here, the pseudo lobule and the necrotic tissue appear to have the most distinct profiles. The PLS-DA result shows a similar trend, with the NT, PL, and cancer being the three most separable sub-regions within the sample (Fig. 3b). However, as seen in Fig. 3c and Additional file 1: Fig. S2c, the NT region possessed a distinct property compared to the PL and cancerous regions as having much less metabolites overall. Its separability seems to result from a simple absence of metabolites (Fig. 3d). The PL and cancerous regions, on the other hand, differed primarily in their metabolite distributions. The heatmap in Fig. 3d shows the overall trend of the metabolite distributions where that of the PL region and of the cancerous region seems to be complementary. Specifically, the metabolites that were more abundant in the cancer region, such as D-alanine were among the least abundant in the PL region. Metabolites such as L-carnitine, which were more abundant in the PL region, were much less abundant in the cancer region. The PL and cancer regions also shared some similarities as they both had an increase in some of the lipids and phospholipids compared to other regions. Particularly, the PL regions showed the highest abundance of phosphocholine molecules such as PC (14:0/20:2(11Z, 14Z)) and PC (22:5(3Z, 7Z, 10Z, 13Z, 16Z)/16:1(9Z)). The fibrosis region shared a similar profile as the PL region, both demonstrated the same trends of change compared to the normal tissues. However, the PL regions showed a progression of such changes. Compared to all the diseased tissues, subregions PC and FT showed highly similar overall metabolic profiles, with PC tissues having higher abundance of a few amino acids, phospholipids, and carboxylic acids.

Having found out all the differential metabolites in each sub-region, a KEGG pathway enrichment plot was constructed to categorize the metabolites in terms of the pathways they are part of. Under the positive ion mode, the most significantly altered pathways as identified by the differential metabolites were the amino acid metabolism pathways such as the beta-alanine metabolism and the arginine and proline metabolism pathways (Fig. 3e). In addition, the choline metabolism in cancer pathway was also significantly altered. Under the negative ion mode, almost all the pathways identified were fatty acid biosynthesis and metabolism pathways (Additional file 1: Fig. S2e).

Lastly, time series analysis was performed on metabolites from all the sub-regions of the HCC sample. The changes in the key metabolites along disease progression were reflected by examining the changes in their distributions in the fibrosis, PL, PC, and cancer regions. Here, differential metabolites with similar trend of changes were grouped into one cluster (Fig. 3f). Certain metabolites, such as d-proline and creatine, showed a general decrease from fibrosis to cancer regions but were particularly high in the PL region. Metabolites such as spermidine, isoleucylproline, deoxyuridine showed continuous decrease from fibrosis to cancer regions.

### Metabolic alterations of non-cancerous regions

**Fig. 4:**
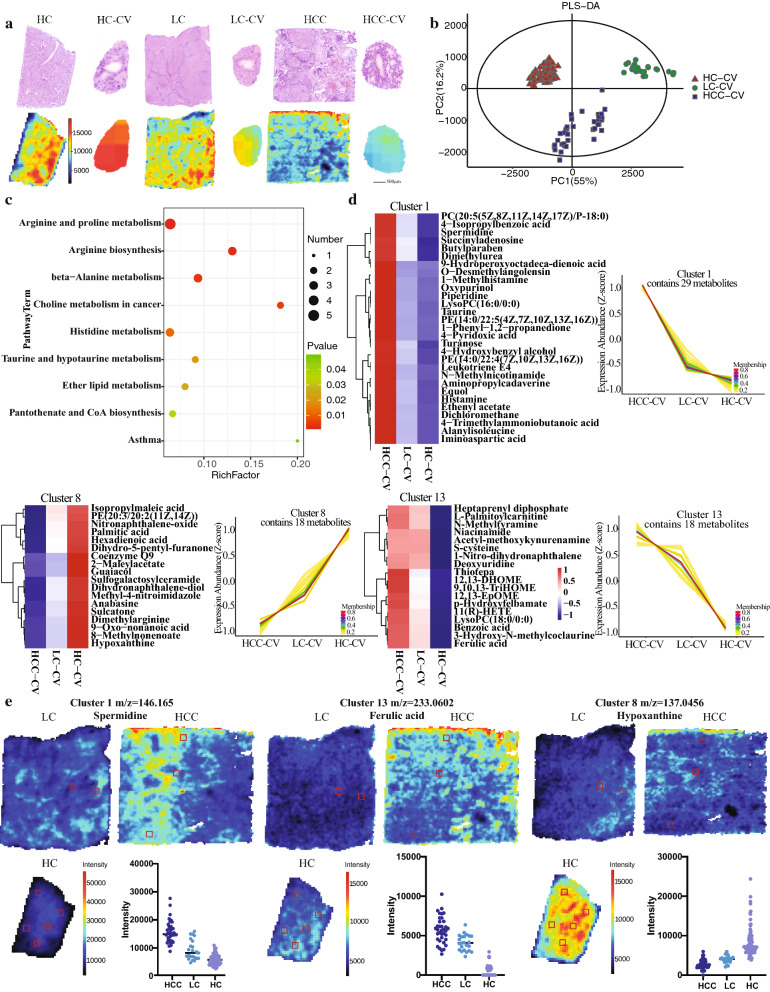
Metabolites’ spatial distributions in the non-cancer regions (CV) of the HC, LC and HCC samples under positive ionization mode. **a** HE and MSI images of the CV regions in the HC, LC and HCC samples. **b** PLS-DA analysis of the CV regions. **c** KEGG analysis of key altered metabolic pathways in the CV non-cancer regions. **d** Sample time series analysis of key metabolite expressions of the non-cancer regions. **e** Examples of key metabolites’ spatial expressions in the CV regions

The central vein regions of all three samples were analyzed separately to see any trend of metabolic alterations within the non-cancerous regions during the disease progression. The H&E and the MSI images reveal the zoomed-in morphology around the central vein region (Fig. 4a). The CV regions of all three tissues appeared to have comparable histology in the H&E stain images. Yet, MS images revealed their different metabolic profiles. PLS-DA analysis and heatmap of metabolites distributions confirmed that the central vein regions in the three samples can be clearly separated (Fig. 4b and c) based on their differential metabolic profiles. The heatmap in Fig. 3c reveals some overall trends of change in metabolite distributions. Many metabolites showed a continuous increase or decrease in abundances from the HC to the LC to the cancer central veins. However, some of the phospholipids and fatty acids were most abundant in the LC central vein and subsequently decreased in intensities in both the cancer and the HC central vein regions. When looking into the specific altered pathways, amino acid and fatty acid biosynthesis and metabolic pathways appeared to be the most altered pathways under positive and negative ion mode, respectively (Fig. 4c and Additional file 1: Fig. S3d). Time series analysis then display some of the characteristic trends of changes in distribution of key metabolites around the central vein region during disease progression. These trends match the ones shown in the heatmap. Numerous metabolites, including spermidine, taurine, histamine, ferulic acid, benzoic acid, and deoxyuridine experienced an overall continuous increase in concentration from the HC central vein to the HCC central vein. Other metabolites, such as isopropylmaleic acid, palmitic acid, and anabasine, showed a reverse trend of continuous decrease in concentration from the HC to HCC central vein (Fig. 4d and e). In Fig. 4e, three typical metabolites were chosen to represent their clusters. The MS images and the quantification plots demonstrate that spermidine (*m/z* 146.165) and ferulic acid (*m/z* 233.0602) increased in intensities in the central vein regions as the disease progress. On the other hand, anabasine (*m/z* 163.1127) continuously decreased in the central vein region. Therefore, non-cancerous regions experience changes in metabolites distribution during pathological changes of the liver.

### Metabolic alterations of cancer-related regions

**Fig. 5:**
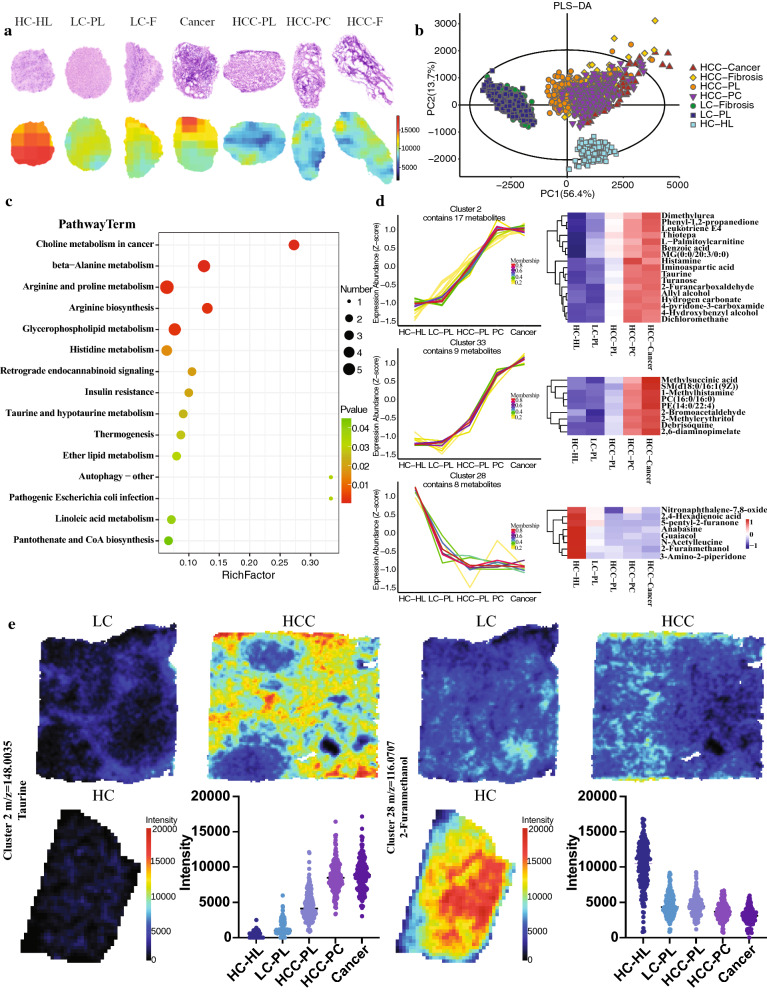
Metabolites’ spatial distributions in the cancer-related regions of the HC, LC and HCC samples under positive ionization mode. **a** HE and MSI images of the cancer-related regions in HC, LC and HCC samples. **b** PLS-DA analysis of the cancer-related regions. **c** KEGG analysis of key altered metabolic pathways in the cancer-related regions. **d** Sample time series analysis of key metabolite expressions in the cancer-related regions. **e** Examples of key metabolites’ spatial expressions in cancer-related regions

All the lesion regions in LC and HCC as well as a healthy control region were examined to show trends of metabolic changes within the diseased areas. First, the H&E and MSI images, heatmap of differential metabolites, along with the PLS-DA confirm the separability of the diseased sub-regions within the three samples based on metabolic profiles and morphologies (Fig. 5a, b). According to the PLS-DA analysis, while the most significant differences existed between the three samples, the sub-regions within the samples also remain separable. Particularly, the fibrosis, PL, PC, and cancer regions of the HCC sample showed variations progressively in one dimension (Fig. 5b). In Fig. 5c, the key metabolic pathways identified from the differential metabolites between the different lesion regions remain largely conserved from previous analysis of the whole tissues. Choline metabolism in cancer, beta-alanine metabolism, arginine and proline metabolism, arginine biosynthesis, and glycerophospholipid metabolism pathways experienced the most alterations. Time series analysis was then conducted following disease progression in such order: HL of HC, PL of LC, PL of HCC, PC of HCC and the cancer region of HCC. Representative metabolite clusters with continuous upward and downward trends are displayed in Fig. 5d. For example, molecules such as taurine (*m/z* 148.0035) has clear continuous increase in concentration from healthy to cancer regions (Fig. 5e). In contrast, metabolites like 2-furanmethanol (*m/z* 116.0707) were found most abundant in healthy lobule of the HC and least abundant in cancer region of the HCC sample. Interestingly, metabolites that showed an upward trend in abundance along disease progression such as the ones in cluster 2 and 33 seemed to experience the sharpest changes from HCC PL to PC areas. On the other hand, cluster 28 represents a downward trend in abundance where the greatest drop occurs between HC and LC tissues.

### ROC analyses of predictive abilities of differential metabolites

**Fig. 6:**
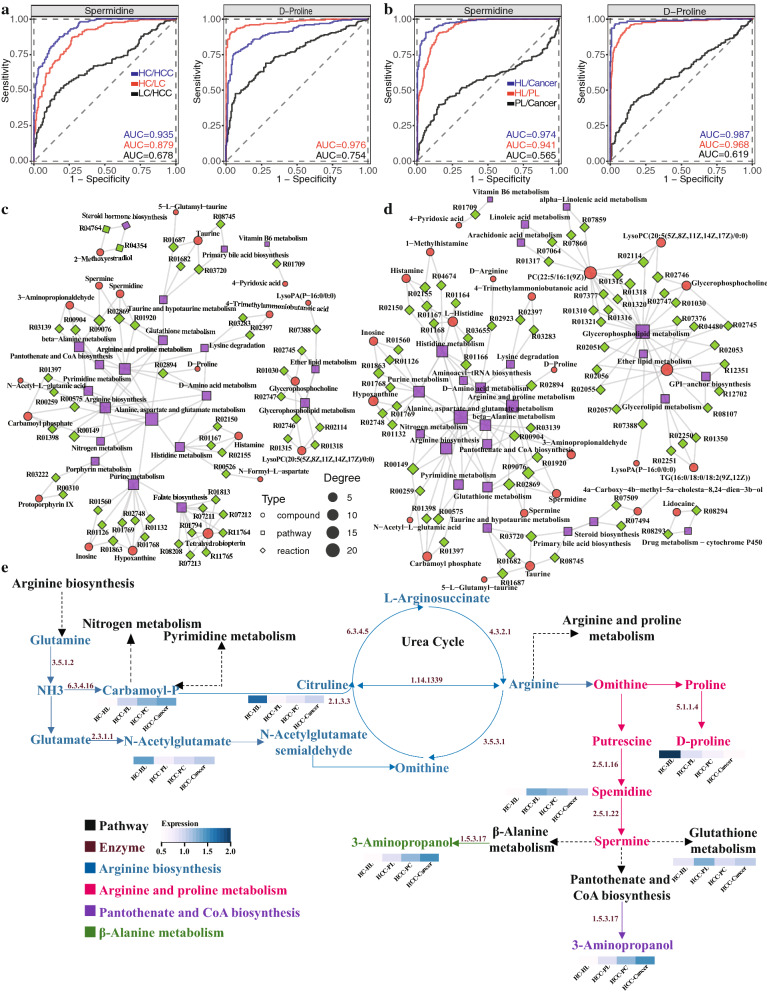
Reconstruction of the key altered metabolic network in the HBV-related liver cirrhosis to liver cancer progression. **a**, **b** ROC analyses of key metabolites at distinguishing between diseased tissues (**a**) and between different subregions (**b**) under positive ionization mode. **c** The network map of key metabolic pathways in the non-cancer regions. **d** The network map of key metabolic pathways in the cancer-related regions. **e** Schematics of the related and altered metabolic pathways in the HBV infection liver disease progression

Receiver Operative Characteristic (ROC) analyses were performed to determine if the metabolites described here can help differentiate between HC, LC, and HCC tissues and even between different subregions of the lesion. According to the plots, the identified differential metabolites under the positive ion mode tend to distinguish healthy from diseased tissues with high accuracy (AUC ~ 0.9) (Fig. 6a; Additional file 1: Fig.S5c). However, the predictive power of the differential metabolites decreases when differentiating between LC and HCC (AUC ~ 0.7). Such decrease in accuracy is probably due to the similar metabolic changes occurring within the LC and HCC tissues. Looking at the subregions, again, the differential metabolites predict HL from cancer region and HL from PL accurately (AUC > 0.97). On the other hand, the differentiation between PL and cancer regions with any single detected metabolite appeared to be challenging as the AUCs are generally around 0.5–0.6 (Fig. 6b).

### Reconstruction of altered metabolic network

Network maps of upregulated metabolites in both the non-cancerous and cancer-related regions were constructed to investigate the key metabolites and metabolic pathways altered in liver cirrhosis to liver cancer progression. For both the non-cancerous and cancer-related regions, amino acid metabolism pathways, especially the arginine and proline metabolism and the alanine, aspartate, and glutamate metabolism, locate in the center of the altered metabolic networks (Fig. 6c and d). Through these pathways, most of the upregulated metabolites were connected. In addition to amino acid metabolism, the glycerophospholipid metabolism pathways connected most of the other upregulated metabolites, particularly in the non-cancerous region. Lastly, taking the key metabolites and metabolic pathways, a schematic of an altered networks in diseased liver tissues was reconstructed (Fig. 6e), which connects amino acid synthesis and metabolism through urea cycle. The changes in intensity are marked for the key altered metabolites along this network. For example, spermidine concentration experienced a continuous decrease from HCC PL to cancer regions. Carbamoyl-P and 3-aminopropanol, on the other hand, showed a continuous increase from the healthy tissues to PL to cancer.

## Discussion

In this study, we conducted ADFADESI-MSI mediated spatial metabolomics to understand the metabolic reprogramming associated with the liver cirrhosis to HCC progression. The use of AFADESI-MSI enabled us to explore the regional heterogeneity of metabolite distributions. With the wide coverage and sensitivity of this MSI technique [12], we were able to detect the spatial distributions of hundreds of metabolites. Then, times series analysis and KEGG pathway enrichment analysis were conducted to identify key altered metabolites and the pathways they are involved in. We discovered that many of the amino acid metabolism and biosynthesis pathways are among the most altered processes in both the cancerous and non-cancerous regions of the diseased liver. Moreover, many of the significantly altered metabolites were also discovered to be associated with the glycerophospholipid metabolism pathways specifically in the diseased tissues.

Before focusing on the diseased tissues of the samples, we examined the surrounding non-cancerous areas to understand the roles of the stromal cells in tumor metabolism. First, some of the well-studied hallmarks of cancer were identified as significantly altered metabolites in our experiment. For example, histamine was found to have a continuous increase in concentrations in the central vein regions as the disease progress. Histamine has previously been identified as one of the factors of tumorigenesis across many types of cancers because of its roles in immune responses, cell proliferations, angiogenesis, etc. [8, 17, 22, 24]. Therefore, the matching results reinforce the validity of our experiment. Furthermore, the transport of the amino acids have been identified as one of the major trades in metabolites between the cancer cells and the surrounding microenvironment [32]. Here, we found that most of the altered metabolites can be associated with amino acid metabolism or biosynthesis. The most altered amino acid metabolism pathways include the beta-alanine metabolism, arginine and proline metabolism, alanine, aspartate and glutamate metabolism, and arginine biosynthesis pathways. Most of these metabolic pathways have been identified as altered pathways in HCC in previous LC-MS/MS studies [2, 6, 14]. However, as most of these studies used LC-MS/MS and looked at serum metabolomics, our results offer additional spatial information regarding the changes of those metabolic processes. The increases in amino acid biosynthesis and metabolism around the cancerous region can be explained by the increased energy-consumption needs of cancer cells. Some amino acids such as glutamate are also important for DNA synthesis. Therefore, their metabolisms in the non-cancerous regions would change to provide additional supply for the cancer cells through bidirectional trades [32]. Other than through the amino acid metabolism pathways, the upregulated metabolites, spermidine, spermine, and 3-aminopropanal, can be linked through a polyamine catabolic enzymatic reaction. In this reaction, spermine can be oxidized into spermidine, and 3-aminopropanal would be converted into hydrogen peroxide (H_2_O_2_), leading to oxidative stress. Therefore, the increase in the spermine oxidase enzyme level has been associated with cancer [5, 23]. The detected changes of the associated metabolite levels may be an indication of the change in spermine oxidase enzyme level. Overall, our results show that the surrounding stroma cells of diseased livers engage in various levels of metabolic reprogramming as the lesion progresses.

More importantly, the areas of cirrhosis and tumor undergo more complete metabolic changes as reflected by their altered metabolites. The pathways that contain the most differential metabolites are still the amino acid metabolism and biosynthesis pathways. A lot of the metabolite changes in the lesion regions are the same as in the non-cancerous regions. For example, both proteinogenic and non-proteinogenic amino acids experienced continuous changes in abundance going from liver cirrhosis to liver cancer. These upregulated amino acids serve to promote protein synthesis, DNA and RNA synthesis, and the conversion into other key metabolites in cancer cells [19, 35]. Therefore, the amino acid metabolisms act as a connection between numerous other metabolic processes, such as the purine and pyrimidine metabolisms and the amnioacyl-tRNA biosynthesis pathways, to fulfill the energy and growth needs for the tumor. In addition, we discovered that the pyrimidine metabolism is also altered through the increase in carbamoyl-phosphate during cancer progression. Carbamoyl-phosphate is derived from ammonia during the first step of the urea cycle. However, the overexpression of the converting enzyme, carbamoyl-phosphate synthetase 1(CPS1), has been found to encourage pyrimidine biosynthesis, which is then connected to tumor proliferation [30]. Since there has been study suggesting the use of CPS1 inhibitor to treat cancer [38], detecting the level of carbamoyl-phosphate, especially with spatial information, could be helpful in finding potential biomarkers or in future drug testing. Another major aspect of metabolic reprogramming is the alteration of the glycerophospholipid metabolism. The changes in this pathway in the cancer-related regions are more significant than that in the non-cancerous regions. Specifically, there are significant increases in some of the phosphatidylcholine (PC), lysophosphatidylcholine (LPC) and glycerophosphocholine levels as the lesion develops. Glycerophospholipid is important in cell membrane formation and thus is commonly upregulated for cell proliferation during cancer development [20, 27]. In addition, multiple studies have associated upregulation of LPC to PC conversion enzyme with various cancers [7, 28, 31]. Furthermore, it is not entirely clear why both the cancer-related and non-cancerous regions saw a continuous increase in some of the antioxidant levels as the disease progress. Particularly, both taurine and ferulic acid are upregulated. Ferulic acid has been shown to be an effective antioxidant and anti-inflammatory compound whose anti-tumor effects have been investigated [33, 34, 36]. Taurine has also been found to have anti-tumor effect by inducing apoptosis [25, 33, 34, 39]. Therefore, it is unlikely that these metabolites are direct metabolites of cancer cells. Rather, the increase of ferulic acid and taurine levels may be an attempt of compensation from the surrounding regions in response to the liver damages. Lastly, our ROC analyses results validate the power of the detected metabolites in differentiating the healthy from diseased tissues. Although using any single metabolite to distinguish liver cirrhosis apart from HCC remains challenging, our results demonstrate the general progressive trends in metabolic changes along disease advancement. Hence, during future studies, it may be possible to explore the combinations of multiple key metabolites in predicting disease progression.

There are still many aspects of metabolic reprogramming to be explored. For example, future studies can work on improving the spatial resolution of the MSI technique so that finer metabolite distribution trends can be analyzed. In addition to the separation of different types of tissues, metabolic changes within each type of tissue can also be examined to help us better understand the sources of metabolic dysregulations. Furthermore, to better understand the causes and effects of the altered metabolic pathways, future studies can conduct multi-omics comparisons. For example, combining spatial metabolomics data with spatial transcriptome data may be able to reveal the relationships between altered gene expressions of relevant enzymes and the metabolomic phenotypes. Another potentially rewarding study is to compare samples that have undergone different therapeutics or diets to understand the suitability and effectiveness of various treatment plans. Overall, though, our study was able to detect many key metabolites that may be able to serve as HCC biomarkers for early detection. Moreover, the time-series analysis and the spatial information provide us a better sense of the metabolic changes along disease progression and across different regions of the liver.

## Supplementary Information

**Additional file 1: Figure S1.** Total metabolite distributions in the HC, LC and HCC tissue samples. **a** HE and MSI diagrams of HC, LC and HCC whole samples under the negative ionization mode. **b** PLS-DA comparison of AFADESI-MSI data under the negative ionization mode. **c**, **d** Alterations of metabolites detected by AFADESI-MSI method in the HC, LC and HCC samples based on positive (**c**) and negative (**d**) ionization modes. **e**, **f** K-means diagrams of the HC, LC and HCC tissue samples based on positive (**e**) and negative (**f**) ionization modes. **Figure S2.** The alterations of metabolites’ spatial distributions in HCC sub-regions under negative ionization mode. **a** HE and MSI images of different sub-regions of HCC. **b** PLS-DA analysis of different HCC sub-regions. **c** Number of metabolites detected from different HCC sub-regions. **d** Heatmap of significantly differentiated metabolites based on VIP > 1. **e** KEGG analysis of key altered metabolic pathways in the sub-regions of HCC. **Figure S3.** Metabolites’ spatial distributions in the non-cancerous regions (CV) in the HC, LC and HCC samples under negative ionization mode. **a** HE and MSI images of the CV regions in the HC, LC and HCC samples. **b** PLS-DA analysis for the CV regions. **c** Heatmap of significantly differentiated metabolites under positive ionization mode in the CV regions based on VIP > 1. **d** KEGG analysis of key altered metabolic pathways in the CV regions. **e** Heatmap of significantly differentiated metabolites based on VIP > 1 under negative ionization mode. **f** Sample time series analysis of the key metabolite expressions in the CV regions. **Figure S4.** Metabolites’ spatial distributions of the cancer-related regions in the HC, LC and HCC samples under negative ionization mode. **a** HE and MSI images (negative mode) of the cancer-related regions in the HC, LC and HCC samples. **b** PLS-DA analysis of the cancer-related regions. **c** Heatmap of significantly differentiated metabolites in the cancer-related regions based on variable VIP > 1 under positive ionization mode. **d** Heatmap of significantly differentiated metabolites in the cancer-related regions based on variable VIP > 1 under negative ionization mode. **e** KEGG analysis of key altered metabolic pathways in cancer-related regions. **Figure S5.** Metabolic changes in different tissues and its predictability of disease progression. **a** Sample time series analysis of key metabolite expressions (negative mode) in the cancer-related regions. **b** Examples of key metabolites’ spatial expressions in cancer-related regions under negative ion mode. **c** ROC analyses of key metabolites at distinguishing between diseased tissues and between different subregions (anabasine was detected under positive ionization mode, docosahexaenoic acid was detected under negative ionization mode).

**Additional file 2: Table S1.** Key parameters of the AFADESI-MSI setting.

## Data Availability

The datasets analyzed during the current study are not publicly available as unpublish data was included but are available from the corresponding author on reasonable request.

## Abbreviations

HCC: Hepatocellular carcinoma
HBV: Hepatitis B virus
MSI: Mass spectrometry imaging
AFADESI: Air flow-assisted desorption electrospray ionization
LC: Liver cirrhosis
ROI: Region of interest
MELD: Model of end stage liver disease
PL: Pseudo lobule
NT: Necrotic tissue
H&E: Hematoxylin and eosin
CPS1: Carbamoyl-phosphate synthetase
LPC: Lysophosphatidylcholine

## Data Availability

The datasets analysed during the current study are not publicly available due to unpublish data was included but are available from the corresponding author on reasonable request.

## References

[1] Bagnardi V, Rota M, Botteri E, Tramacere I, Islami F, Fedirko V, Scotti L, Jenab M, Turati F, Pasquali E, Pelucchi C, Galeone C, Bellocco R, Negri E, Corrao G, Boffetta P, La Vecchia C (2015). Alcohol consumption and site-specific cancer risk: a comprehensive dose–response meta-analysis. Br J Cancer.

[2] Baniasadi H, Gowda GAN, Gu H, Zeng A, Zhuang S, Skill N, Maluccio M, Raftery D (2013). Targeted metabolic profiling of hepatocellular carcinoma and hepatitis C using LC-MS/MS. Electrophoresis.

[3] Capurro MI, Xiang Y-Y, Lobe C, Filmus J (2005). Glypican-3 promotes the growth of hepatocellular carcinoma by stimulating canonical wnt signaling. Cancer Res.

[4] Cech NB, Enke CG (2001). Practical implications of some recent studies in electrospray ionization fundamentals. Mass Spectrom Rev.

[5] Chaturvedi R, de Sablet T, Peek RM, Wilson KT (2012). Spermine oxidase, a polyamine catabolic enzyme that links Helicobacter pylori CagA and gastric cancer risk. Gut Microbes.

[6] Chen T, Xie G, Wang X, Fan J, Qiu Y, Zheng X, Qi X, Cao Y, Su M, Wang X, Xu LX, Yen Y, Liu P, Jia W (2011). Serum and urine metabolite profiling reveals potential biomarkers of human hepatocellular carcinoma. Mol Cell Proteomics MCP.

[7] Du Y, Wang Q, Zhang X, Wang X, Qin C, Sheng Z, Yin H, Jiang C, Li J, Xu T (2017). Lysophosphatidylcholine acyltransferase 1 upregulation and concomitant phospholipid alterations in clear cell renal cell carcinoma. J Exp Clin Cancer Res.

[8] Fang Z, Yao W, Xiong Y, Li J, Liu L, Shi L, Zhang W, Zhang C, Nie L, Wan J (2011). Attenuated expression of HRH4 in colorectal carcinomas: a potential influence on tumor growth and progression. BMC Cancer.

[9] Ferrarini A, Di Poto C, He S, Tu C, Varghese RS, Balla AK, Jayatilake M, Li Z, Ghaffari K, Fan Z, Sherif ZA, Kumar D, Kroemer A, Tadesse MG, Ressom HW (2019). Metabolomic analysis of liver tissues for characterization of hepatocellular carcinoma. J Proteome Res.

[10] Ferreri C, Sansone A, Ferreri R, Amézaga J, Tueros I (2020). Fatty acids and membrane lipidomics in oncology: a cross-road of nutritional, signaling and metabolic pathways. Metabolites.

[11] Haybaeck J, Zeller N, Wolf MJ, Weber A, Wagner U, Kurrer MO, Bremer J, Iezzi G, Graf R, Clavien P-A, Thimme R, Blum H, Nedospasov SA, Zatloukal K, Ramzan M, Ciesek S, Pietschmann T, Marche PN, Karin M, Kopf M, Browning JL, Aguzzi A, Heikenwalder M (2009). A lymphotoxin-driven pathway to hepatocellular carcinoma. Cancer Cell.

[12] He J, Sun C, Li T, Luo Z, Huang L, Song X, Li X, Abliz Z (2018). A sensitive and wide coverage ambient mass spectrometry imaging method for functional metabolites based molecular histology. Adv Sci.

[13] Higashitsuji H, Higashitsuji H, Itoh K, Sakurai T, Nagao T, Sumitomo Y, Sumitomo H, Masuda T, Dawson S, Shimada Y, Mayer RJ, Fujita J (2005). The oncoprotein gankyrin binds to MDM2/HDM2, enhancing ubiquitylation and degradation of p53. Cancer Cell.

[14] Huang Q, Tan Y, Yin P, Ye G, Gao P, Lu X, Wang H, Xu G (2013). Metabolic characterization of hepatocellular carcinoma using nontargeted tissue metabolomics. Cancer Res.

[15] Jinjuvadia R, Patel S, Liangpunsakul S (2014). The association between metabolic syndrome and hepatocellular carcinoma: systemic review and meta-analysis. J Clin Gastroenterol.

[16] Kang HJ, Chung DH, Sung CO, Yoo SH, Yu E, Kim N, Lee SH, Song JY, Kim CJ, Choi J (2017). SHP2 is induced by the HBx-NF-κB pathway and contributes to fibrosis during human early hepatocellular carcinoma development. Oncotarget.

[17] Kennedy L, Hodges K, Meng F, Alpini G, Francis H (2012). Histamine and histamine receptor regulation of gastrointestinal cancers. Transl Gastrointest Cancer.

[18] Li L, Wang H (2016). Heterogeneity of liver cancer and personalized therapy. Cancer Lett.

[19] Li Z, Zhang H (2016). Reprogramming of glucose, fatty acid and amino acid metabolism for cancer progression. Cell Mol Life Sci.

[20] Liu Y, Hong Z, Tan G, Dong X, Yang G, Zhao L, Chen X, Zhu Z, Lou Z, Qian B, Zhang G, Chai Y (2014). NMR and LC/MS-based global metabolomics to identify serum biomarkers differentiating hepatocellular carcinoma from liver cirrhosis. Int J Cancer.

[21] McGlynn KA, Petrick JL, El-Serag HB (2021). Epidemiology of hepatocellular carcinoma. Hepatol Baltim Md.

[22] Moya-García AA, Pino-Ángeles A, Sánchez-Jiménez F, Urdiales JL, Medina MÁ (2021). Histamine, metabolic remodelling and angiogenesis: a systems level approach. Biomolecules.

[23] Murray Stewart T, Dunston TT, Woster PM, Casero RA (2018). Polyamine catabolism and oxidative damage. J Biol Chem.

[24] Nguyen PL, Cho J (2021). Pathophysiological roles of histamine receptors in cancer progression: implications and perspectives as potential molecular targets. Biomolecules.

[25] Park S-H, Lee H, Park KK, Kim HW, Park T (2006). Taurine-responsive genes related to signal transduction as identified by cDNA microarray analyses of HepG2 cells. J Med Food.

[26] Rao W, Pan N, Tian X, Yang Z (2016). High-resolution ambient MS imaging of negative ions in positive ion mode: using dicationic reagents with the single-probe. J Am Soc Mass Spectrom.

[27] Santos CR, Schulze A (2012). Lipid metabolism in cancer. FEBS J.

[28] Shida-Sakazume T, Endo-Sakamoto Y, Unozawa M, Fukumoto C, Shimada K, Kasamatsu A, Ogawara K, Yokoe H, Shiiba M, Tanzawa H, Uzawa K (2015). Lysophosphatidylcholine acyltransferase1 overexpression promotes oral squamous cell carcinoma progression via enhanced biosynthesis of platelet-activating factor. PLoS ONE.

[29] Singh AK, Kumar R, Pandey AK (2018). Hepatocellular carcinoma: causes, mechanism of progression and biomarkers. Curr Chem Genomics Transl Med.

[30] Taguchi A, Fahrmann JF, Hanash SM (2020). A promising CPS1 inhibitor keeping ammonia from fueling cancer. Cell Chem Biol.

[31] Uehara T, Kikuchi H, Miyazaki S, Iino I, Setoguchi T, Hiramatsu Y, Ohta M, Kamiya K, Morita Y, Tanaka H, Baba S, Hayasaka T, Setou M, Konno H (2016). Overexpression of lysophosphatidylcholine acyltransferase 1 and concomitant lipid alterations in gastric cancer. Ann Surg Oncol.

[32] Vettore L, Westbrook RL, Tennant DA (2020). New aspects of amino acid metabolism in cancer. Br J Cancer.

[33] Wang AS, Lodi A, Rivera LB, Izquierdo-Garcia JL, Firpo MA, Mulvihill SJ, Tempero MA, Bergers G, Ronen SM (2014). HR-MAS MRS of the pancreas reveals reduced lipid and elevated lactate and taurine associated with early pancreatic cancer. NMR Biomed.

[34] Wang J, Sheng Y, Ji L, Wang Z (2014). Ferulic acid prevents liver injury and increases the anti-tumor effect of diosbulbin B in vivo. J Zhejiang Univ Sci B.

[35] Wei Z, Liu X, Cheng C, Yu W, Yi P (2021). Metabolism of amino acids in cancer. Front Cell Dev Biol.

[36] Wu J, Xue X, Fan G, Gu Y, Zhou F, Zheng Q, Liu R, Li Y, Ma B, Li S, Huang G, Ma L, Li X (2021). Ferulic acid ameliorates hepatic inflammation and fibrotic liver injury by inhibiting PTP1B activity and subsequent promoting AMPK phosphorylation. Front Pharmacol.

[37] Xia Y, Cheng X, Li Y, Valdez K, Chen W, Liang TJ (2018). Hepatitis B virus deregulates the cell cycle to promote viral replication and a premalignant phenotype. J Virol.

[38] Yao S, Nguyen T-V, Rolfe A, Agrawal AA, Ke J, Peng S, Colombo F, Yu S, Bouchard P, Wu J, Huang K-C, Bao X, Omoto K, Selvaraj A, Yu L, Ioannidis S, Vaillancourt FH, Zhu P, Larsen NA, Bolduc DM (2020). Small molecule inhibition of CPS1 activity through an allosteric pocket. Cell Chem Biol.

[39] Zhang X, Tu S, Wang Y, Xu B, Wan F (2014). Mechanism of taurine-induced apoptosis in human colon cancer cells. Acta Biochim Biophys Sin.

