# Supplementary figures and images for: Spatial metabolomics on liver cirrhosis to hepatocellular carcinoma progression
Source: Cancer Cell Int. 2022 Nov 24;22:366. doi: 10.1186/s12935-022-02775-9 (PMC9686114; doi:10.1186/s12935-022-02775-9)

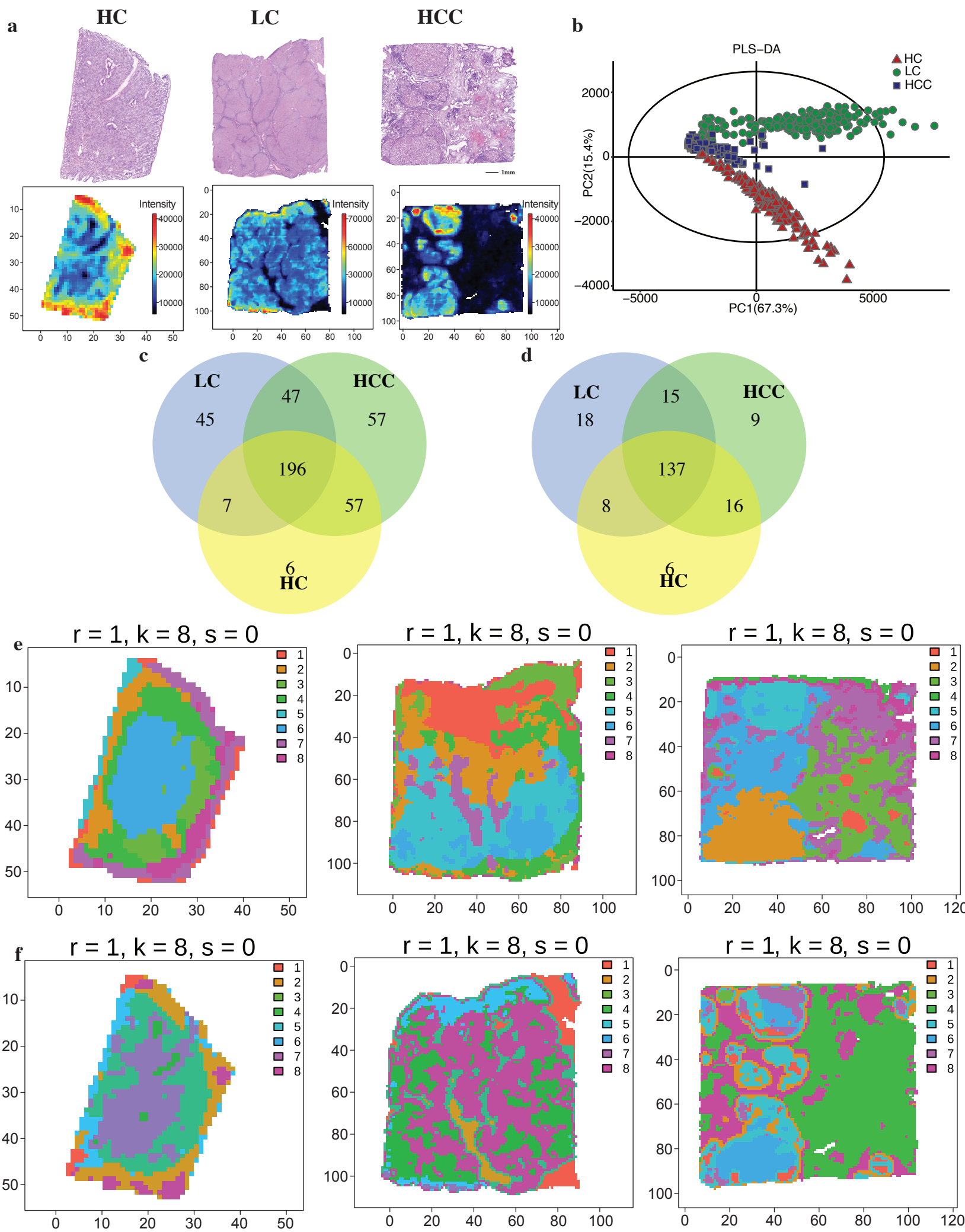

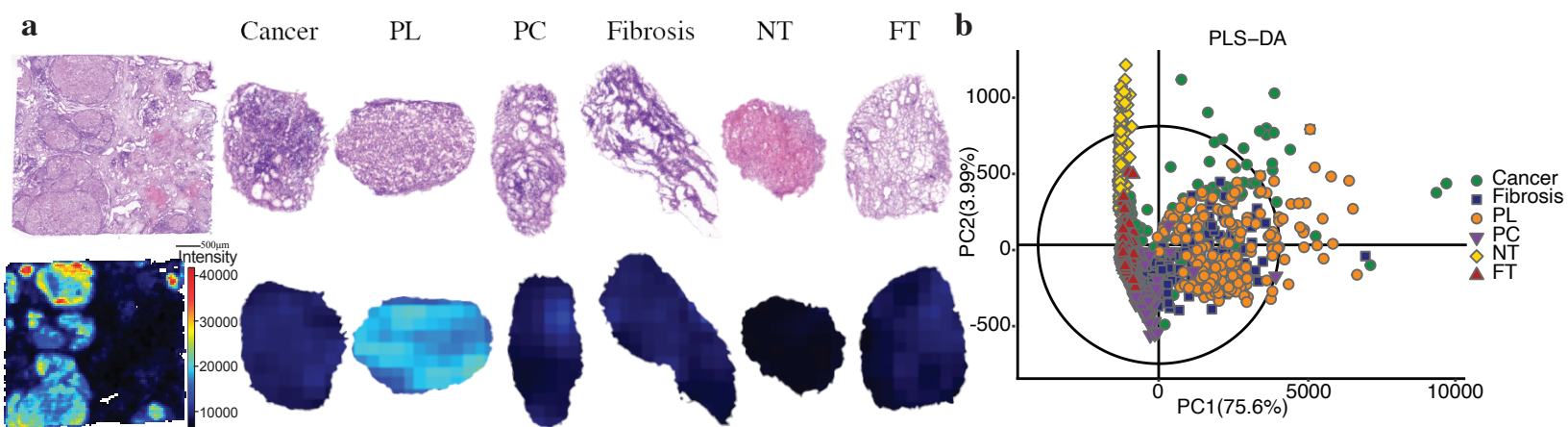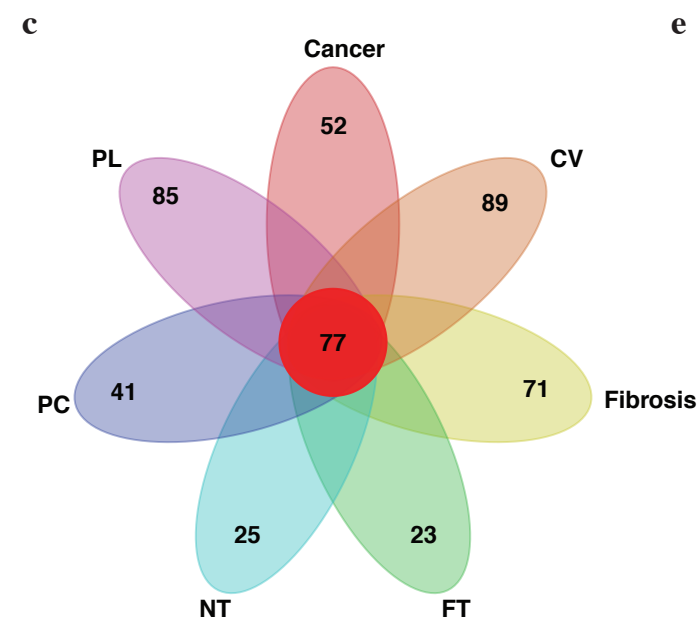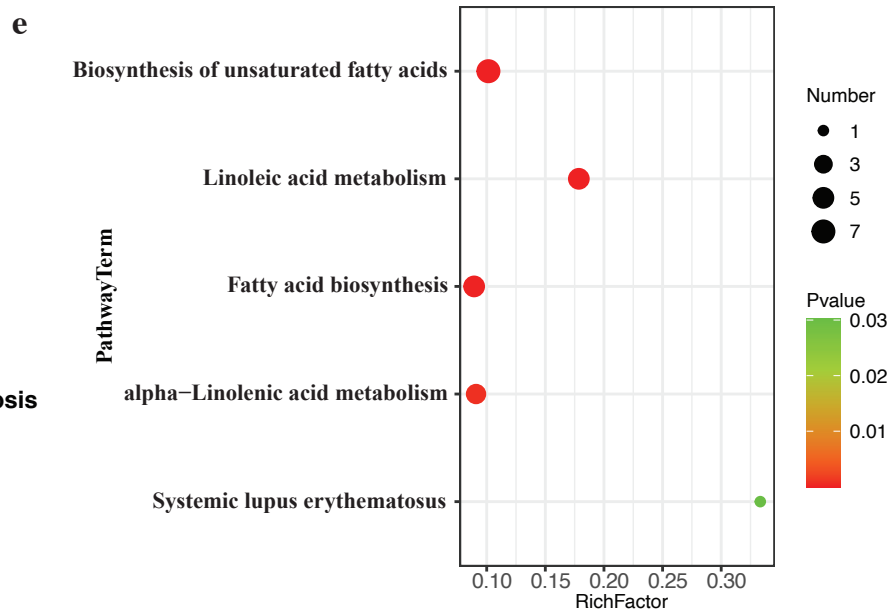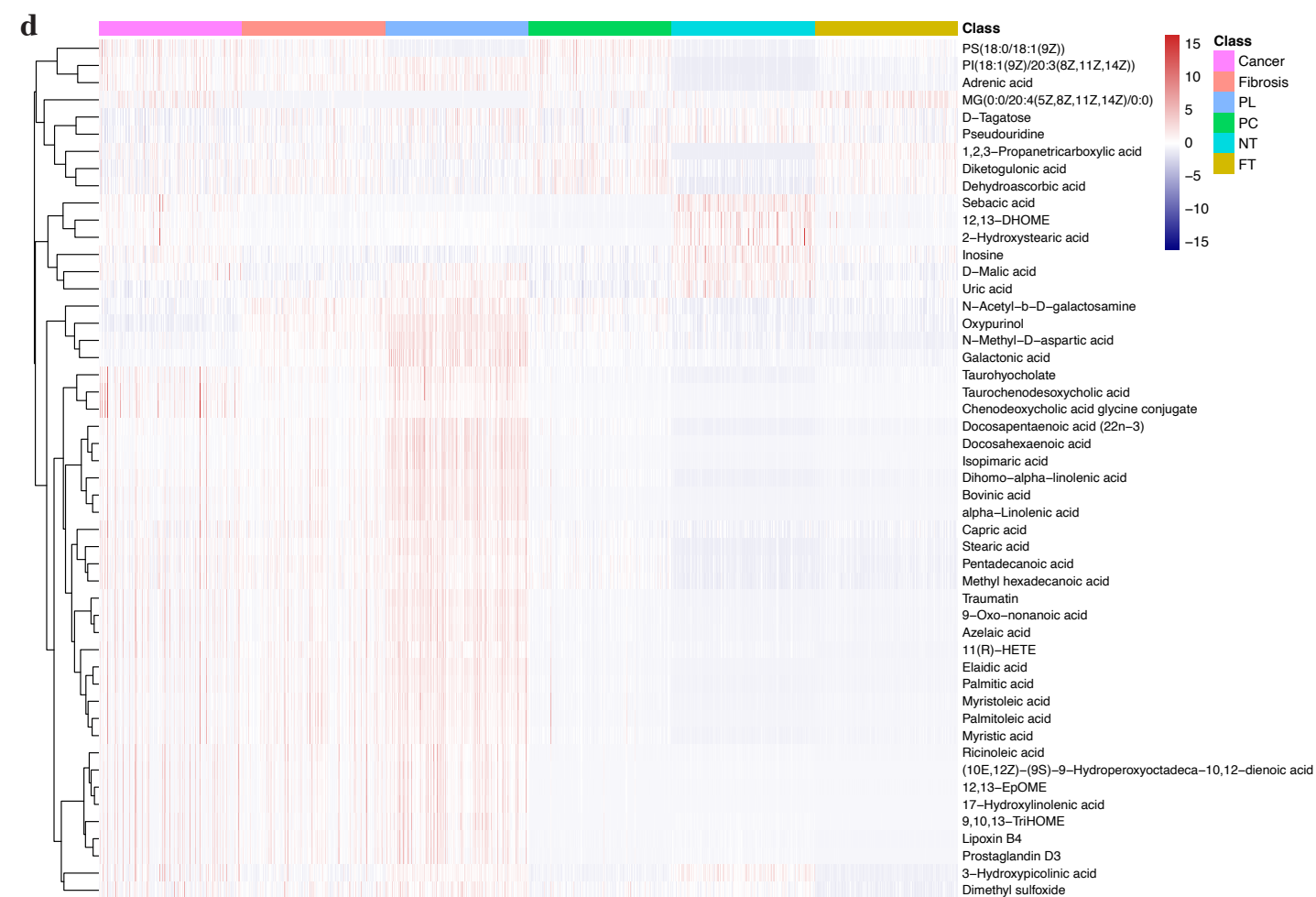

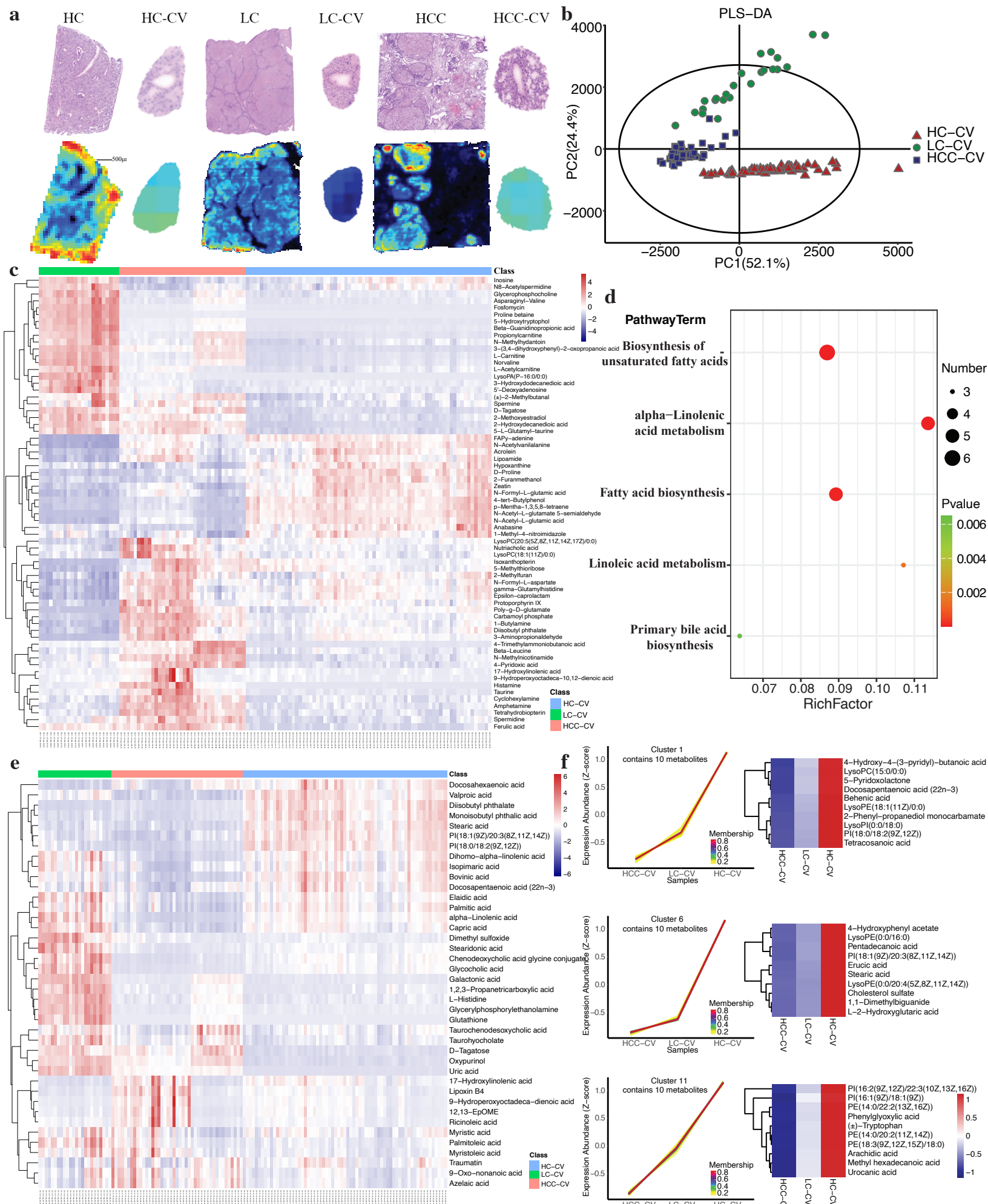

**a**

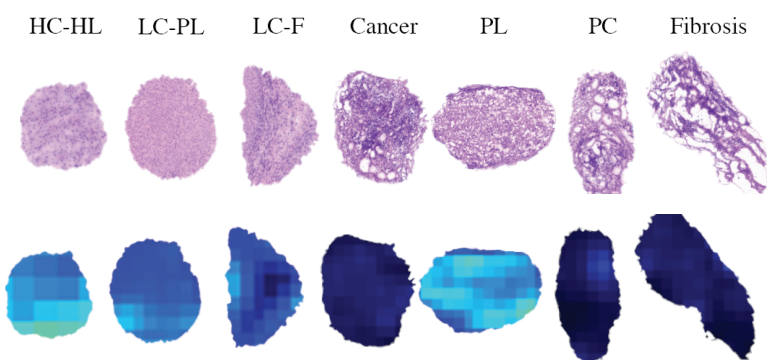

**b**

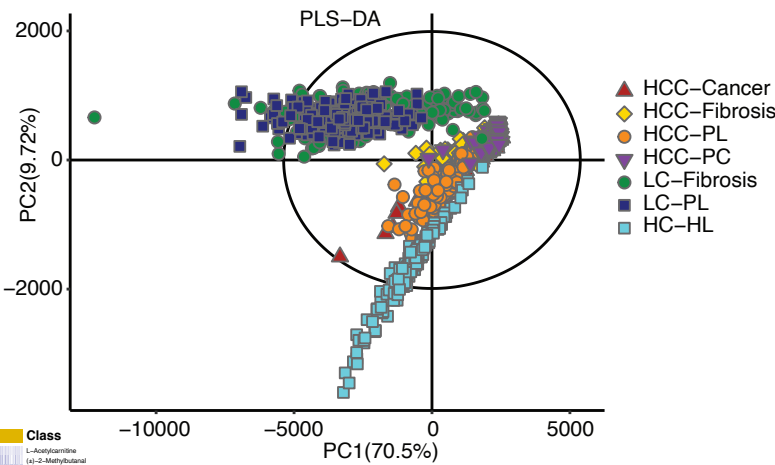

**c**

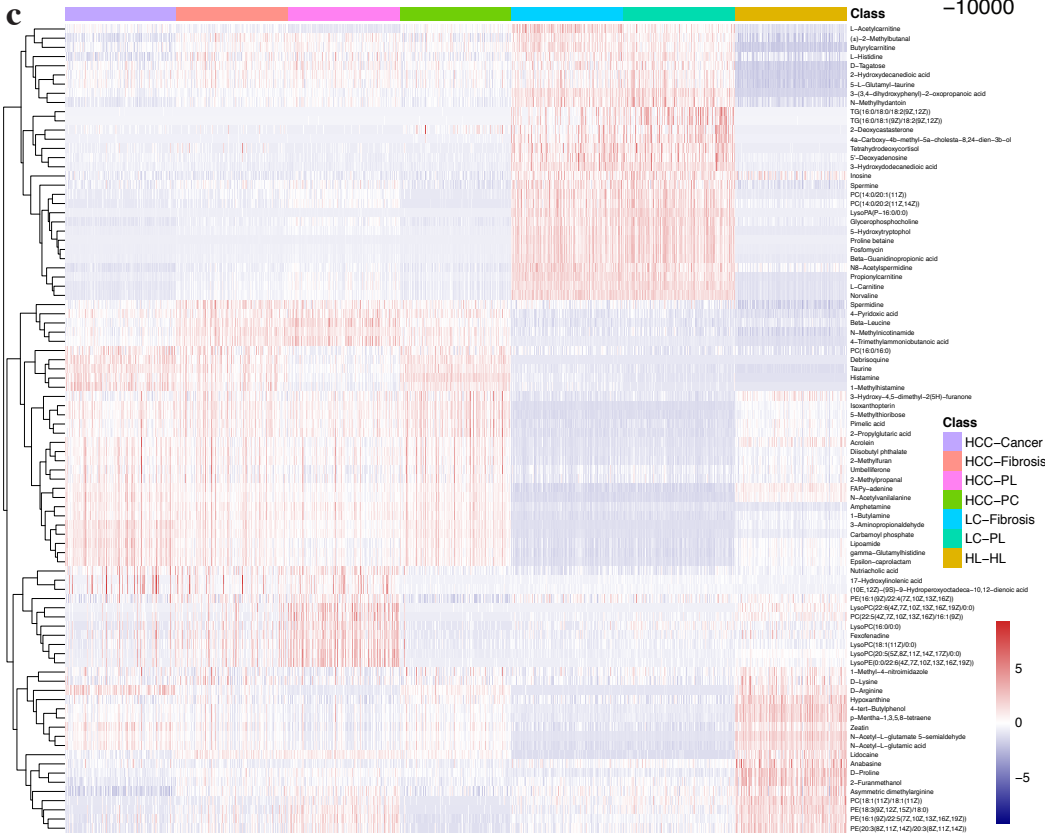

**d**

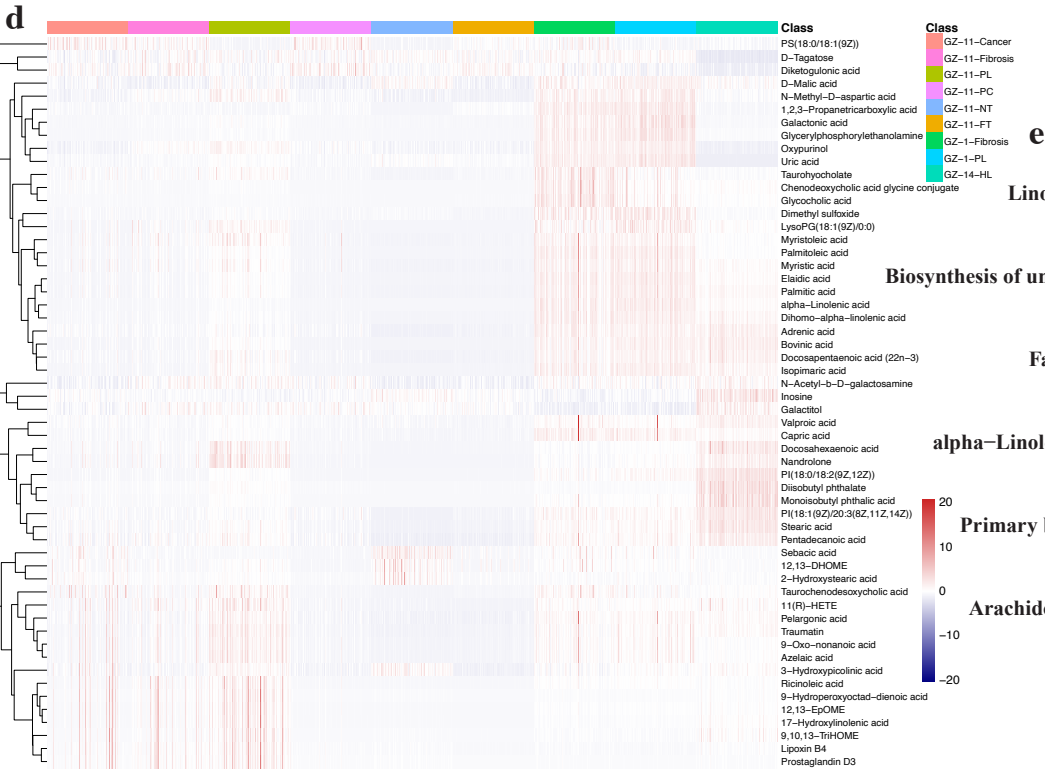

**e**

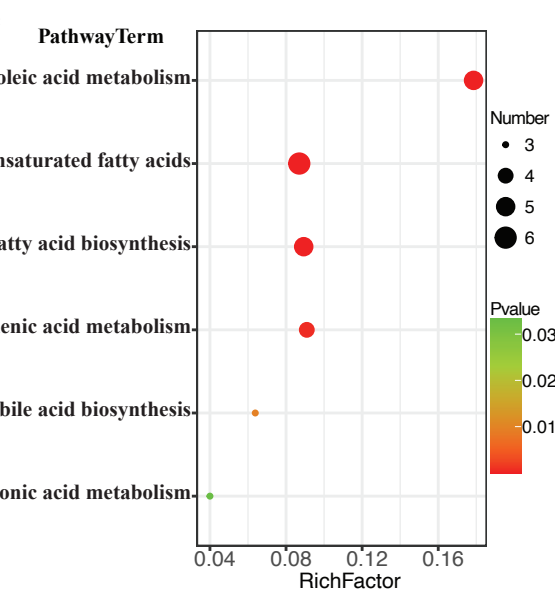

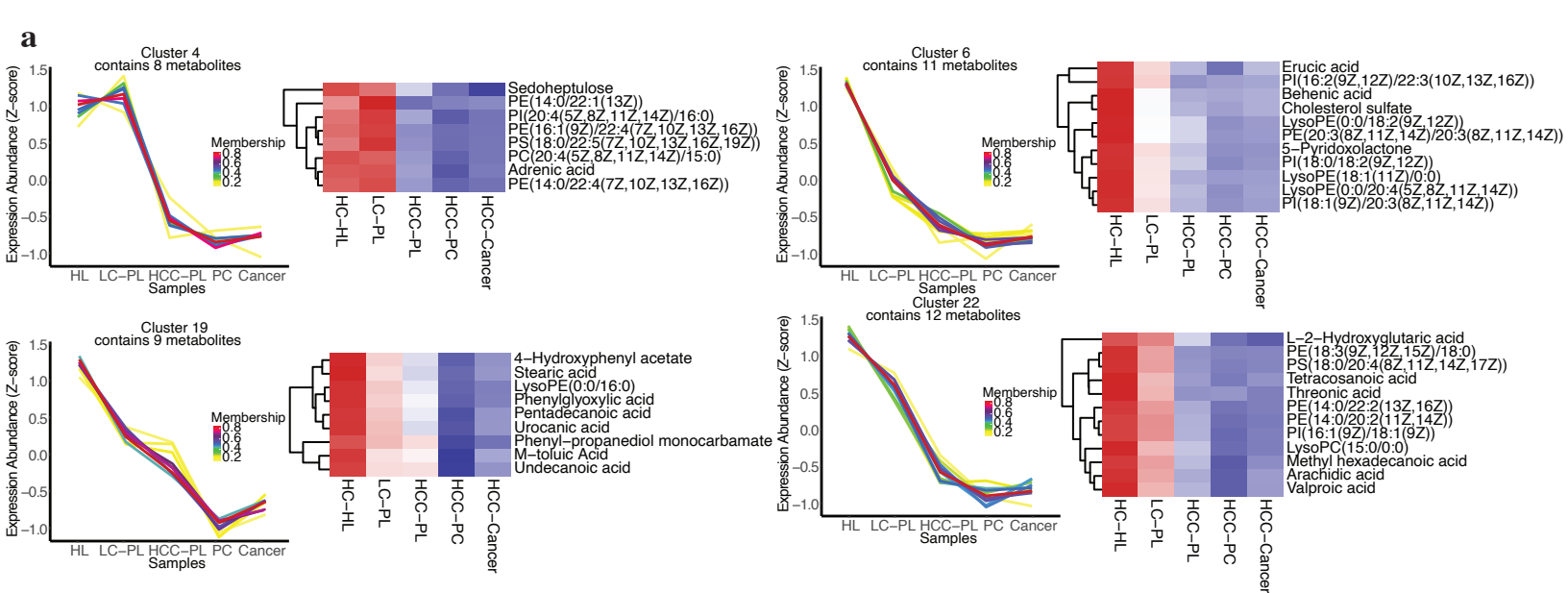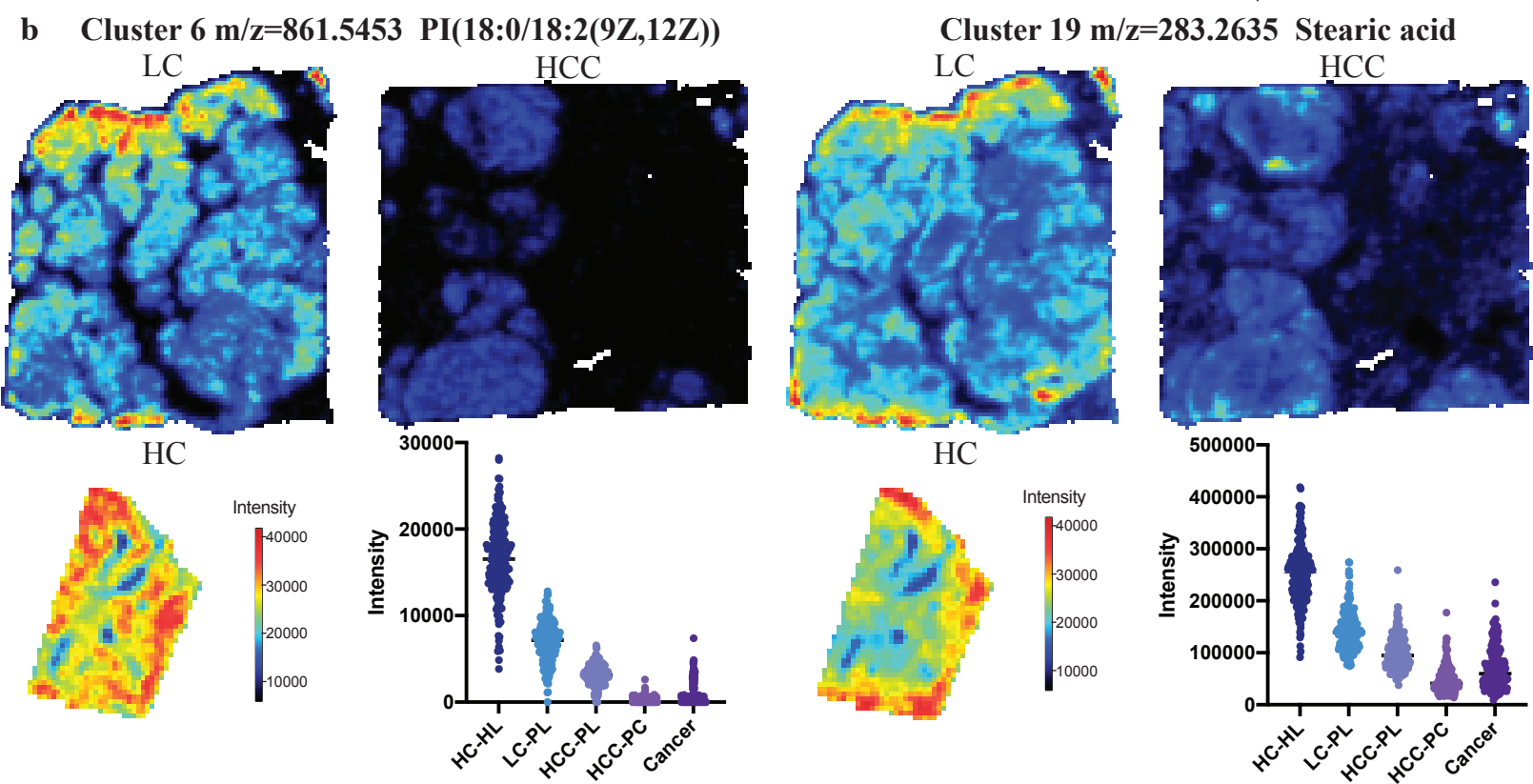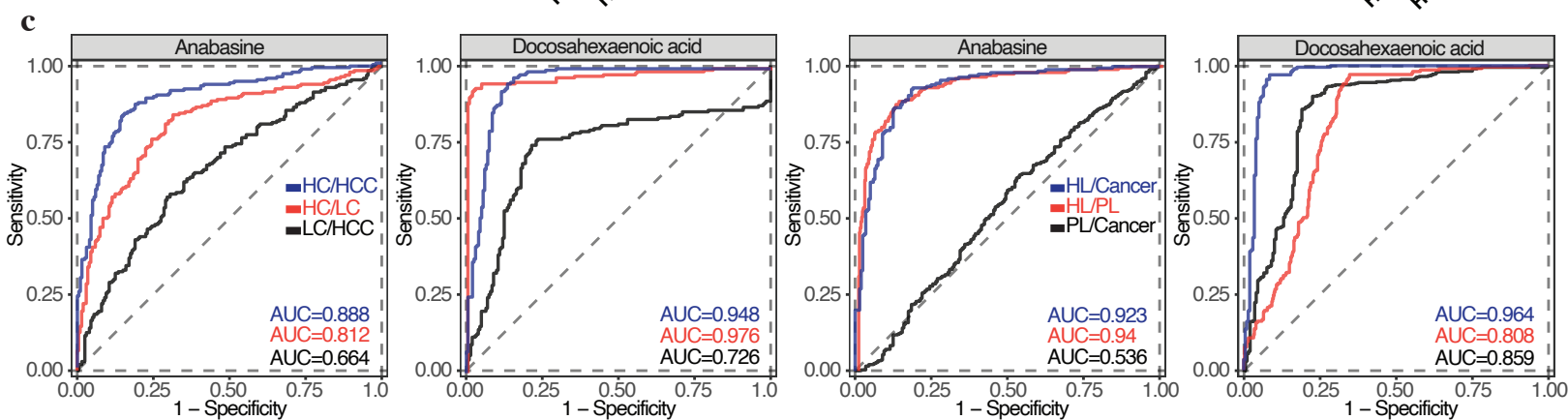

Supplement: Supplementary file 1 — Additional file 1: Figure S1. Total metabolite distributions in the HC, LC and HCC tissue samples. a HE and MSI diagrams of HC, LC and HCC whole samples under the negative ionization mode. b PLS-DA comparison of AFADESI-MSI data under the negative ionization mode. c, d Alterations of metabolites detected by AFADESI-MSI method in the HC, LC and HCC samples based on positive (c) and negative (d) ionization modes. e, f K-means diagrams of the HC, LC and HCC tissue samples based on positive (e) and negative (f) ionization modes. Figure S2. The alterations of metabolites’ spatial distributions in HCC sub-regions under negative ionization mode. a HE and MSI images of different sub-regions of HCC. b PLS-DA analysis of different HCC sub-regions. c Number of metabolites detected from different HCC sub-regions. d Heatmap of significantly differentiated metabolites based on VIP > 1. e KEGG analysis of key altered metabolic pathways in the sub-regions of HCC. Figure S3. Metabolites’ spatial distributions in the non-cancerous regions (CV) in the HC, LC and HCC samples under negative ionization mode. a HE and MSI images of the CV regions in the HC, LC and HCC samples. b PLS-DA analysis for the CV regions. c Heatmap of significantly differentiated metabolites under positive ionization mode in the CV regions based on VIP > 1. d KEGG analysis of key altered metabolic pathways in the CV regions. e Heatmap of significantly differentiated metabolites based on VIP > 1 under negative ionization mode. f Sample time series analysis of the key metabolite expressions in the CV regions. Figure S4. Metabolites’ spatial distributions of the cancer-related regions in the HC, LC and HCC samples under negative ionization mode. a HE and MSI images (negative mode) of the cancer-related regions in the HC, LC and HCC samples. b PLS-DA analysis of the cancer-related regions. c Heatmap of significantly differentiated metabolites in the cancer-related regions based on variable VIP > 1 under positive ioni [file 12935_2022_2775_MOESM1_ESM.pdf]
