# Supplementary material for: Spatial metabolomics on liver cirrhosis to hepatocellular carcinoma progression
Source: Cancer Cell Int. 2022 Nov 24;22:366. doi: 10.1186/s12935-022-02775-9 (PMC9686114; doi:10.1186/s12935-022-02775-9)
Supplement: Supplementary file 2 — Additional file 2: Table S1. Key parameters of the AFADESI-MSI setting. [file 12935_2022_2775_MOESM2_ESM.docx]

Table S1. Key parameters of the AFADESI-MSI setting.

| **Parameters** | **Values** |
| --- | --- |
| Vx (mm/s) | 0.2 |
| Dy (mm) | 0.1 |
| Dt (s) | 7 |
| X-axis length (mm) | 10 |
| Y-axis length (mm) | 10 |
| Spray voltage (V) | ±0 |
| Guide tube voltage (V) | ±0 |
| Sheath gas flow rate (L/min) | 0 |
| Aux gas flow rate (L/min) | 0 |
| Sweep gas flow rate (L/min) | 0 |
| Capillary temperature (°C) | 350 |
| Aux gas heater temperature (°C) | 0 |
| Scan mode | Full MS |
| Scan range (Da) | 100-1000 |
| Resolution | 70000 |
| Spray gas press (MPa) | 0.6 |
| Spray angle | 60° |
| Distance from sprayer to surface (mm) | 0.7 |
| Distance from sprayer to guide tube (mm) | 3 |
| Distance from orifice to guide tube (mm) | 10 |
| Extracting gas flow rate (L/min) | 45 |
